# A Feasibility Study of Trans-Arterial Chemoembolization as a First-Line Treatment for Locally Advanced Breast Cancer

**DOI:** 10.1007/s00270-026-04471-x

**Published:** 2026-05-14

**Authors:** Ryusuke Kuzumi, Shinichi Hori, Atsushi Hori, Masato Takeuchi, Ikuo Dejima, Tatsuya Nakamura

**Affiliations:** 1 Department of Interventional Radiology, Institute for Image Guided Therapy, Osaka, Japan; 2 Department of Minimum Invasive Therapy, Rakuwakai Otowa Hospital, Kyoto, Japan

**Keywords:** Breast cancer, Locally advanced breast cancer, TACE, Transarterial treatment, Microcatheter, Spherical embolic material

## Abstract

**Purpose:**

To evaluate the feasibility of transarterial chemoembolization (TACE) for locally advanced breast cancer (LABC).

**Materials and Methods:**

A total of 31 sequential patients with previously untreated LABC in Stages III or IV were evaluated retrospectively. Selective microcatheter insertion was performed on all branches angiographically identified to the breast tumours and axillary lymph node metastases. Angio-CT imaging was used to confirm the distribution of drugs within the tumour after the selective infusion of contrast material. The patients were treated with anti-neoplastic agents indicated for breast cancer therapy. Drugs were infused directly into the tumour-feeding arteries, and embolization was carried out with spherical embolic material, HepaSphere (50–100 µm). After the initial treatment, additional treatments were repeated as needed. The primary outcome measure was the local response of the primary tumours and lymph node metastases at 1, 3, 6, and 12 months after the initial therapy.

**Results:**

The mean number of sessions per patient was 6.8 (range 3–23). The response rates of primary breast lesions at 1, 2, 6, and 12 months were 19%, 48%, 79%, and 70%, respectively. No major adverse events requiring therapy were reported. Local pain, bleeding, or infection were well controlled after the initial treatment. A minor complication of treatment was a skin reaction characterised by discolouration. The 1 and 2-year survival rates were 87.1% and 67.6%, respectively. The overall survival was 29.7 months. The prognostic factor most strongly affecting survival was distant metastasis.

**Conclusion:**

TACE is a safe and effective first-line treatment for LABC.

**Supplementary Information:**

The online version contains supplementary material available at https://doi.org/10.1007/s00270-026-04471-x.

## Introduction

It is estimated that 5–10% of newly diagnosed breast cancer patients worldwide present with de novo Stage IV cancer [1, 2]. In patients with locally advanced breast cancer (LABC), according to breast cancer treatment guidelines [3], systemic chemotherapy should be introduced first, followed by total mastectomy and postoperative radiation therapy, and then systemic chemotherapy should be continued to prevent local recurrence and control of systemic metastases. However, patients sometimes fear the side effects of systemic chemotherapy, expect breast preservation, and are often reluctant to accept the treatment outlined in the guidelines. In addition, the circumstances of highly advanced cancer may make treatment inapplicable, or patients may have comorbidities that preclude the introduction of treatment. Medical, psychological, and social aspects should be carefully considered when planning treatment [4]. Due to the development of guiding catheters and microcatheters, transarterial approaches have become safer and more reliable in recent years. The use of diagnostic imaging techniques such as cone-beam computed tomography (CT) or angio-CT has been implemented during treatment. A variety of embolic materials have been improved to reduce adverse events, and spherical embolic agents can now be used for the treatment of breast cancer. Despite substantial progress in systemic pharmacotherapy, the effectiveness of standard therapy in LABC remains suboptimal, particularly with respect to local disease control. A transarterial treatment method, such as arterial chemoinfusion [5, 6] or TACE [7–9], has been considered a treatment option for LABC because of the limitations of systemic chemotherapy. It is necessary to demonstrate that this treatment is effective even in previously untreated LABC cases. The aim of this study was to evaluate the feasibility of transarterial treatment with transarterial chemoembolization (TACE) as a first-line treatment for LABC.

## Materials and Methods

### Study Design and Setting

We analysed a single-centre retrospective observation cohort of consecutive patients treated between November 2016 and December 2022 at an interventional oncology service. Follow-up continued until December 31, 2023. The Institutional Review Board approved the study (protocol2023-24); informed consent for the procedures was obtained.

### Patients

The inclusion criteria were as follows: (1) histologically confirmed breast carcinoma; (2) de novo LABC defined as T3 (> 5 cm) or T4 (chest wall/skin invasion) with/without ipsilateral axillary adenopathy; (3) no prior oncologic therapy for the index cancer; and (4) planned management with TACE as first-line locoregional therapy. The exclusion criteria were as follows: (1) prior systemic therapy, surgery, or radiotherapy for the index tumour; (2) uncontrolled coagulopathy; (3) severe contrast allergy; and (4) Eastern Cooperative Oncology Group (ECOG) performance status < 2.

### Diagnostic Imaging

The main diagnostic modality for evaluating the size of tumours is an enhancing mass in the breast tissue on contrast-enhanced CT. Three-dimensional images of all branches of the subclavian artery to the breast and axillary regions were reconstructed from preoperative dynamic CT scans and/or angio-CT during the procedure in all patients. Distant metastases were evaluated by CT, MRI or positron emission tomography‒CT.

### Treatment Method

In all cases, treatment was performed in the rooms of hybrid angio-CT suites (Toshiba, Infinix, Tokyo, Japan; Canon Alfinix, Tokyo, Japan). A 4 Fr 30 cm-long sheath (Terumo, Tokyo, Japan) was inserted into the abdominal aorta through the femoral artery. A digital subtraction angiography (DSA) image of the subclavian artery on the side of the breast tumour was obtained using a 4 Fr diagnostic catheter (injection rate/volume; 1.5–2.0 mL/sec/7.5–10.0 mL). Angio-CT of the subclavian artery (injection rate/volume; 1.2 mL/sec/7.5–15.0 mL) was used for the reconstruction of 3D images of the subclavian artery and its branches when sufficient information could not be obtained from the 3D images from the pre-procedure CT scan. Using DSA and 3D images, a reshaped 2.0 Fr microcatheter (Estram IGT model, Tory Medical, Tokyo, Japan) was advanced into all supplying arteries without microguidewire guidance to avoid arterial spasm, which may result in inadequate drug distribution at the target site. The internal thoracic artery, thoracoacromial artery, lateral thoracic artery, subscapular trunk, and circumflex mammary artery were targeted. Angio-CTs during contrast injection (0.2–0.4 ml/sec/2.0–4.0 mL) into all the supplying arteries were performed to evaluate the extent of the enhanced area of the tumour to confirm the blood supply to the tumour. According to the tumour volume enhanced by the selective angio-CT, the dose of the antineoplastic agents for each tumour-feeding artery was allocated. Anti-neoplastic agents were administered via intra-arterial infusion at a slow rate of approximately 0.1 mL/sec to prevent overflow from the target artery. Anti-neoplastic agents, including anthracyclines (20–30 mg), docetaxel (20 mg), mitomycin (4–6 mg), cisplatin (20–30 mg), and fluorouracil (250–500 mg), are standard drugs for treating breast cancer. Anthracyclines are usually combined with fluorouracil or mitomycin. Docetaxel is combined with cisplatin due to low local irritability caused by these drugs. If patients wished to avoid alopecia, doxorubicin hydrochloride (10–20 mg) was selected; if patients were concerned about skin reactions, docetaxel was selected as the main drug. Almost all patients received bevacizumab (100–200 mg), except those with active tumour bleeding. Each drug was diluted with saline to a volume of 20 mL. The dose of the antineoplastic agent for each session was determined by the total volume of each patient’s tumour and was not adjusted for body weight. The total dose of drugs used was substantially lower than that used in systemic chemotherapy. No patients received systemic chemotherapy during the study period. Immediately after the infusion of each drug, embolization using a superabsorbent microsphere, HepaSphere (50–100 µm) (Merit Medical Systems, South Jordan, Utah), was performed. Epirubicin (20 mg) was dissolved in 4 ml of non-ionic contrast material with 1 ml of 10% NaCl, and the solution was injected into a vial of HepaSphere cells (25 mg) and left for 20 min. With this method, the size of the HepaSphere microspheres ranged from 100 to 200 µm. The other drug loaded was 250 mg of fluorouracil, which was injected into a vial of HepaSpheres (50–100 µm) with 5 ml of solvent and left for 20 min. The HepaSphere size ranged from 200–300 µm. The endpoint of embolization was not complete cessation of blood flow but flow reduction under fluoroscopic observation.

After the initial treatment, a follow-up CT examination was performed to evaluate the effectiveness of the treatment for the breast tumour and the axillary lymph node metastases within 4 weeks. When tumour reduction and/or improvement of symptoms were confirmed, the same treatment was repeated. When it was not effective, treatment with other drugs was performed. After three sessions of treatment, additional treatments with the same procedure were scheduled when the tumours progressed.

The primary outcome measures were the regression rates of both primary tumours and axillary lymph node metastases and adverse events. Primary tumours and axillary lymph node metastases were measured on enhanced CT at 1, 3, 6, and 12 months after the initial treatment. According to the Response Evaluation Criteria in Solid Tumours (RECIST) criteria (Ver. 1.1), the local response was evaluated. Based on the RECIST guidelines, the summation of the longitudinal axis of up to two lesions was evaluated for the primary tumours. For the lymph node metastases, axes shorter than 15 mm were measured according to the RECIST criteria. Adverse events during the hospital stay and one month after treatment were surveyed according to Common Terminology Criteria for Adverse Events (CTCAE) Ver5.

The secondary outcome measure was overall survival. Survival time was defined as the interval from the initial date of transarterial treatment to the most recent follow-up date. The Kaplan‒Meier estimated the survival curves, and the prognostic factors were evaluated by the log-rank test, in which a *p* value less than 0.05 was considered statistically significant.

## Results

Thirty-one patients with LABC underwent transarterial chemoembolization. Baseline characteristics are summarised in Table 1. The mean age of the patients was 59.4 (40–81) years. Among 31 patients, 26 had ipsilateral axillary adenopathy, and 12 had distant metastases. The mean hospital stay per session was 3.3 days (range 3–4). The mean number of sessions per patient was 6.8 (range 3–23). No patients were lost to follow-up before the first post-treatment assessment.

**Table 1:** Patient characteristics

Patients’ characteristics	Primary tumour T3 (larger than 5 cm)	Primary tumour T4 (chest wall and/or skin invasion)
Number of patients	5	26
Age (Average)	46–62(52.0)	40–81 (60.4)
Ipsilateral axillary lymph node metastases	5	21
Primary tumour size		
< 5	–	9
5–10	3	8
10 <	1	3
Distant metastases	1	11
Lung and mediastinum	1	6
Liver	1	6
Bone	0	3
Performance Status (ECOG)		
0	3	5
1	2	19
2	0	1
3	0	0
Receptors		
Oestrogen receptor ( +)	3	11
Progesterone receptor ( +)	3	3
HER 2 protein (3 +)	1	7
Triple negative	2	5

All primary lesions and axillary lymph node metastases were successfully treated, microcatheters were inserted into all the tumour-related arteries, and TACE was performed adequately. The arteries treated are shown in Table 2.

**Table 2:** Treatment characteristics (first treatment session)

Treatment characteristics	Primary breast tumours and axillary lymph node metastases
Treated arterial branches	
Internal thoracic artery	31
Lateral thoracic artery	25
Subscapular trunk	19
Thoraco-acromial artery	13
Circumflex mammary artery	10
Average number of treated arteries	3.2
Anti-neoplastic agents	
Anthracycline (10–20 mg)	28
Docetaxel (10–20 mg)	12
Fluorouracil (250 mg)	30
Cisplatin (10–20 mg)	7
Carboplatin(20–30 mg)	9
Mitomycin (2–4 mg)	10
Bevacizumab (100–200 mg)	9
Drugs loaded on SAP-MS*	
Fluorouracil	14
Adriamycin or Epirubicin	16
None	1

SAP-MS*: Superabsorbent polymer microsphere (HepaSphere)

The most common main drug used was anthracycline in the first session of treatment. In the second session, the drugs were changed according to the local effects on the breast tumours. The drugs loaded on the HepaSphere microspheres were adriamycin or fluorouracil in the first session (Table 2).

The reduction rates in the diameter of primary breast tumours were 19.1% (1 month), 34.2% (3 months), 41.4% (6 months), and 40.0% (12 months). The local response according to RECIST is described in Table 3. All the cases were controlled with partial remission (PR) or stable disease (SD) until 6 months later. The disease control rate was 88% at one year (Table 3).

**Table 3:** Treatment response of primary breast tumours

	1 month	3 months	6 months	12 months
CR	0/31	0/31	0/24	0/17
PR	6/31 (19%)	15/31 (48%)	19/24 (79%)	12/17 (70%)
SD	25/31 (81%)	16/31 (52%)	5/24 (21%)	3/17 (18%)
PD	0/31	0/31	0/24	2/17 (12%)
Average reduction rate	19.1%	34.2%	41.4%	40.0%

CR: Complete remission, PR: Partial remission, SD: Stable disease, PD: Progressive disease

The reduction rates of axillary lymph node metastases were 19.0% (1 month), 31.5% (3 months), 37.6% (6 months), and 45.4% (12 months), respectively (Table 4). The time course of the lymph node response was similar to that of the primary tumour. The most common adverse event was local pain after treatment; however, it was treated with oral analgesics. Although tumour bleeding decreased with treatment, slight bleeding from the tumour was observed in four cases during the hospital stay. However, the bleeding was controlled with manual compression. Drug-induced allergic reactions were identified, and the patients were treated with medication. Skin reactions, i.e., skin redness, were observed in 6 patients and were mainly caused by anthracyclines (Table 5). One month after treatment, local pain in 3 patients and tumour bleeding in 2 patients was easily treated. Pigmentation of the breast and axillary region was found in 2 patients (Table 6). In the blood tests, there was no significant change after the treatments, except for the changes in the levels of tumour markers.

**Table 4:** Treatment response of patients with axillary lymph node metastases

	1 month	3 months	6 months	12 months
CR	0/23	0/23	0/20	0/15
PR	5/23 (23%)	14/23 (61%)	15/20 (75%)	11/15 (73%)
SD	18/23 (78%)	9/23 (39%)	5/20 (25%)	4/15 (27%)
PD	0/23	0/23	0/20	0/15
Average reduction rate	19.0%	31.5%	37.6%	45.4%

Twenty-three patients whose lymph nodes were larger than 15 mm in the shorter axis were evaluated for lymph node metastases according to the RESCIST criteria. CR; complete remission, PR; partial remission, SD; stable disease, PD; progressive disease

**Table 5:** Adverse events during the hospital stay in 31 patients

Adverse events	G1	G2	G3	G4
Local pain	*22*	1	0	0
Tumour bleeding	4	0	0	0
Allergic reaction	3	1	0	0
Skin reaction	6	0	0	0

Adverse effect grading was performed according to the classification of the CTCAE Ver. 5

**Table 6:** Adverse events after one month in 31 patients

Adverse events	G1	G2	G3	G4
Local pain	*3*	0	0	0
Tumour bleeding	2	0	0	0
Allergic reaction	0	0	0	0
Skin pigmentation	2	0	0	0

**Fig. 1:**
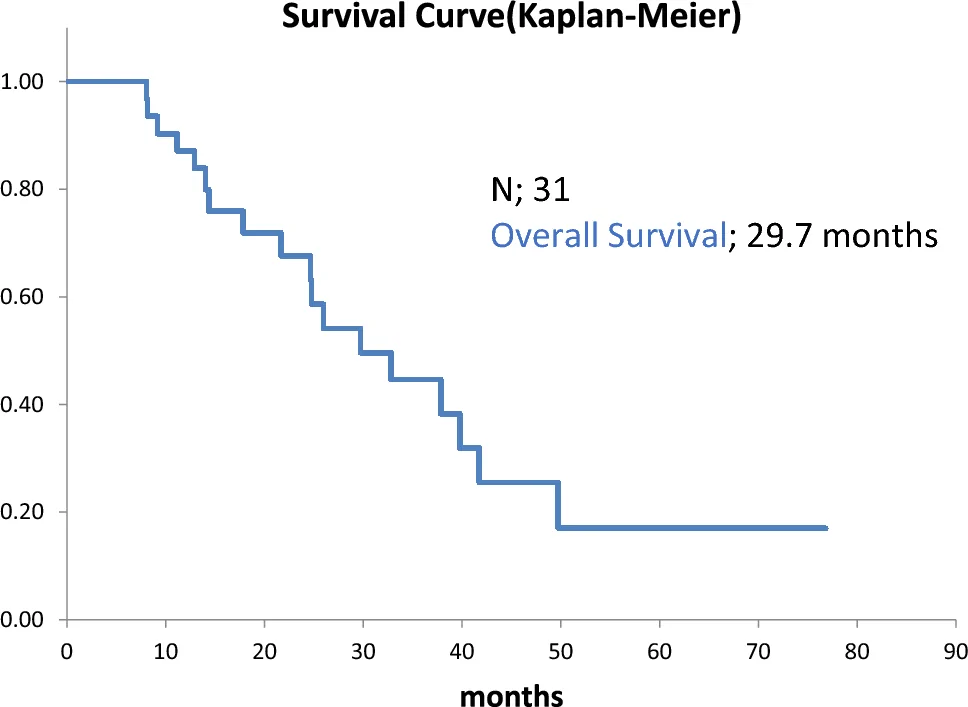
Survival curves of 31 patients with Stages III and IV disease

**Fig. 2:**
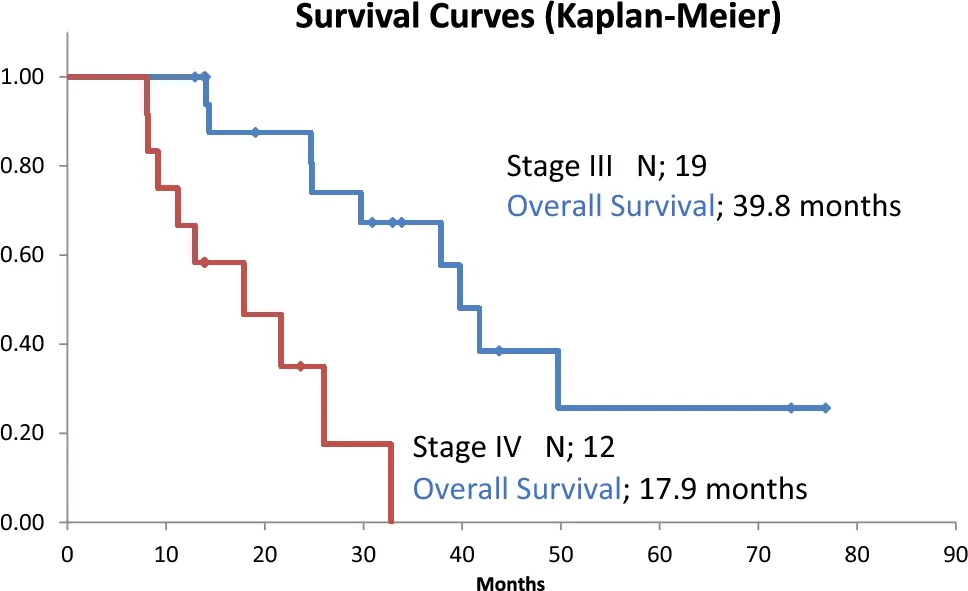
Survival curves of stage III (blue line) and stage IV (red line) patients

**Fig. 3:**
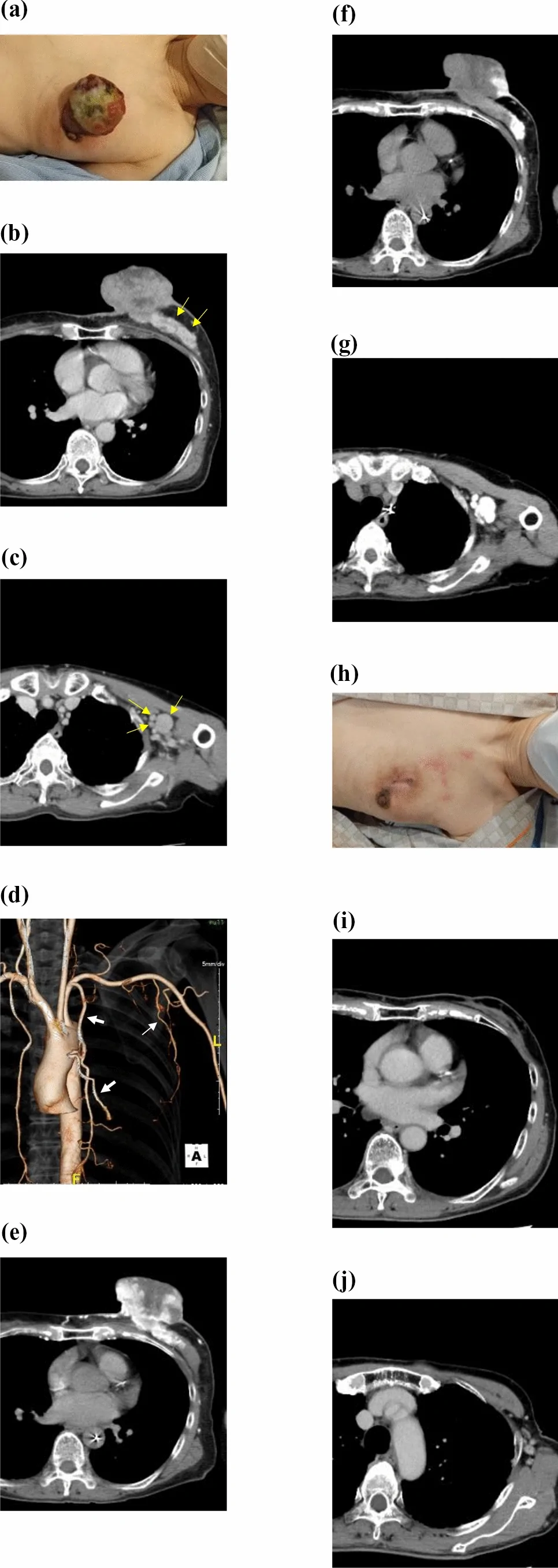
A 67-year-old woman with T4N1M0 primary breast cancer in a de novo setting. **a** A 7 × 5 cm protruding tumour with active bleeding, pain, and infection. **b** The breast tumour on the left side, which was markedly enhanced by contrast enhancement (arrows), had a protruding component. **c** Lymph node metastases were also detected as enhanced nodules (arrows) in the left axilla. **d** 3D images of the subclavian artery reconstructed from an enhanced CT study. Large arrows; left internal thoracic artery and its dilated mammary branch. Small arrow; left lateral thoracic artery. **e** An angio-CT image of the left internal thoracic artery. Contrast agent (6.0 mL) was injected at a rate of 0.6 mL/sec. A large part of the protruding tumour and the medial part of the breast tumour were enhanced. **f** An angio-CT image of the left lateral thoracic artery. The lateral part of the protruding tumour and the lateral margin of the breast tumour were enhanced. **g** An agio-CT image of the left lateral thoracic artery. Axillary lymph node metastases were markedly enhanced. **h** One year after treatment initiation. A total of 6 treatment sessions were repeated. The tumour site was re-epithelialised. Several spotty skin reactions are likely caused by anti-neoplastic agents. **i** There was no evident tumour in the primary site after one year. **j** The size of the lymph nodes in the axilla markedly decreased after one year. They are all within normal size

The one-year survival and two-year survival rates were 87.1% and 67.6%, respectively. The overall survival (OS) was 29.7 months (Fig. 1). The OS times of Stage 3 and Stage 4 patients were 39.8 and 17.9 months, respectively, which were significantly different (*P* < 0.001; log-rank test) (Fig. 2). There was no significant difference between the HER2( +) and HER2(−) groups (*P* = 0.19). A typical case and its clinical course are shown in Fig. 3.

## Discussion

Despite increased public awareness, it is still common to see patients who present with LABC. Many patients with LABC have symptoms such as local pain, bleeding from ulcerative lesions, or tumour infection [4]. An effective treatment is urgently needed to improve their symptoms. The guidelines recommend standard therapy [3]; however, systemic toxicity remains high, with significant risks of cardiotoxicity or immune suppression as well as a high incidence of local recurrence after surgery or radiotherapy. Alternatively, arterial infusion chemotherapy has been used as a neoadjuvant therapy for LABC since the 1960s [5]. Anti-neoplastic agents are administered to the tumour after surgical cannulation through the internal thoracic and subclavian arteries [5, 6, 10–12]. With advances in catheter techniques, percutaneous transarterial administration has been introduced, yielding better clinical results [13–19]. Compared with systemic chemotherapy, regional chemotherapy attempts to deliver higher doses of chemotherapeutic agents to the tumour, directly perfusing the tumour mass via its arterial supply [20–22]. Embolization of the tumour-supplying artery immediately after infusion of anti-neoplastic agents was combined to help retain drugs within tumours, resulting in better local control of LABC [8, 22–24].

After selective catheter insertion, a CT scan along with selective contrast injection is a reliable method to confirm the arterial supply to the tumours and to allocate the dose of anticancer drugs to each artery [25, 26]. For this reason, an angio-suite combined with cone-beam CT function or an angio-CT hybrid system is desirable for safe and efficient treatment.

Since the initial report by Yamada, TACE has become a standard method for treating hepatocellular carcinoma [27]. However, it has seldom been introduced for the treatment of extrahepatic malignancies for fear of serious complications such as skin necrosis caused by embolic material, which is a combination of Lipiodol and gelatine sponges[28]. Damage to skin or normal tissues is a major concern for maintaining the quality of life (QoL) of patients. On the other hand, HepaSphere microspheres, which are larger than 50 microns, cannot occlude capillary circulation in the skin or normal organs. In fact, no patients developed skin necrosis after the embolization of breast tumours in this study. In this treatment, the purpose of embolization using HepaSphere microspheres was not to achieve ischaemic effects but to achieve sufficient retention of antineoplastic agents inside targeted tumours. The reason for using HepaSphere cells was that they possess drug-release capabilities and can exert long-term anti-tumour effects.

The major blood supply to the medial aspect of the breast arises from the perforating branches of the internal mammary artery. The lateral aspect is variably supplied by branches from the subclavian artery [14, 17, 20, 29]. The lateral thoracic artery is the main artery, but there are many anatomical variations. In one-third of patients, breast tumours are supplied from a serpiginous vessel from the brachial artery, which is an uncommon but not rare circulation to the breast tissue [17, 22, 30].

In previous reports [11, 17, 20, 29], a dye was infused to confirm blood circulation to the tumour area. Immediately after infusion, the discolouration of the skin was checked to estimate the feeding area of each tumour-related artery. This method is not reliable because it involves observing skin discolouration but not the distribution of dye in breast tumours. In this study, angio-CTs were carried out in all the patients to determine the blood supply to the target tumours and lymph node metastases.

The main blood supply to the axillary lymph node metastases was the lateral thoracic and/or thoracodorsal arteries.

The drugs used in this study were standard drugs for treating breast cancer. The proposed main drugs were anthracycline or taxane. In previous studies [11, 12, 14, 18], anthracyclines (doxorubicin, epirubicin, and mitoxiantrone) were commonly used, but they caused skin reactions and pigmentation [4, 14, 20]. Recently, taxanes have become standard drugs [21], but they may cause alopecia or immunosuppression even if at low doses. Small doses of cisplatin (10–20 mg), fluorouracil (250 mg), irinotecan (20–40 mg), mitomycin (2–4 mg), or bevacizumab (100–200 mg), all standard drugs for treating breast cancer, were combined. If the reduction in tumour volume was not adequate or allergic reactions were detected during the evaluation one month after treatment, the drugs were switched to others. The choice and optimal dose of anticancer drugs need to be clarified in future studies. It may also be necessary to adopt a method of selecting the most appropriate drug through gene panel testing.

Comparisons of treatment results with various reports were difficult because the patients’ backgrounds, treatment strategies, and evaluations differed. Without the combination of other treatments, the control rates after 6 months and 12 months were 100% and 88%, respectively. The survival benefit in this study was comparable to that in previous reports [2, 20, 22]. The one-year and two-year survival rates were 87.1% and 67.6%, respectively. Even though a cure cannot be expected with this treatment, achieving local control is important.

Only a few reports [8, 23, 31, 32] have described axillary lymph node metastases, even though they are very important from a clinical perspective. More than 90% of LABC cases are associated with regional lymph node metastases [21, 24], a finding observed in 87% of cases in this study. Axillary lymph node metastases were also supplied by branches from the subclavian artery as primary breast lesions. Successful control of axillary lymph node metastases was achieved without serious complications, as in primary tumours.

Side effects were almost negligible, except for a mild skin rash caused by anthracyclines for a few days after treatment [4, 8, 20, 21]. Moreover, pigmentation lasting a couple of months was sometimes cosmetically problematic. Some complications, such as pain, upper limb atrophy, or headache, have been reported [24, 31] when high doses of anti-cancer drugs are used. The low incidence of adverse events in this study is probably due to the low drug doses administered. In the trial using Callisphere, embolization was completed through complete eradication of the supplying artery. On the other hand, embolization was terminated when the blood flow became slow in this study. The local irritability of HepaSphere microspheres, which have high elasticity [33], should be investigated and compared with that of other solid microspheres in clinical studies.

This study is retrospective but not comparative. Multi-institutional comparative studies between TACE and systemic chemotherapy for LABC are necessary. More precise analyses of not only histopathological information but also molecular factors are necessary. The proper drugs, combinations, and doses for intra-arterial administration also need to be clarified in future studies.

## Conclusion

On the basis of our results, we conclude that TACE for primary locally advanced breast cancer can be regarded as feasible, safe and effective.

## Supplementary Information

Below is the link to the electronic supplementary material.

Supplementary file1 (PPTX 1143 KB)
